# On the Relation between Bursts and Dynamic Synapse Properties: a Modulation-Based Ansatz

**DOI:** 10.1155/2009/658474

**Published:** 2009-06-25

**Authors:** Christian Mayr, Johannes Partzsch, Rene Schüffny

**Affiliations:** Chair for Parallel VLSI Systems and Neural Circuits, Dresden University of Technology, 01062 Dresden, Germany

## Abstract

When entering a synapse, presynaptic pulse trains are filtered according to the recent pulse history at the synapse and also with respect to their own pulse time course. Various behavioral models have tried to reproduce these complex filtering properties. In particular, the quantal model of neurotransmitter release has been shown to be highly selective for particular presynaptic pulse patterns. However, since the original, pulse-iterative quantal model does not lend itself to mathematical analysis, investigations have only been carried out via simulations. In contrast, we derive a comprehensive explicit expression for the quantal model. We show the correlation between the parameters of this explicit expression and the preferred spike train pattern of the synapse. In particular, our analysis of the transmission of modulated pulse trains across a dynamic synapse links the original parameters of the quantal model to the transmission efficacy of two major spiking regimes, that is, bursting and constant-rate ones.

## 1. Introduction

The main computational function of artificial neural networks has traditionally been modeled as an adjustment of the coupling weight between neurons. In biological nets, this coupling weight is provided by the synapse, where an incoming (presynaptic) pulse causes a release of neurotransmitters, which in turn generate a postsynaptic current (PSC) that charges the postsynaptic (i.e., receiving) neuron membrane [1]. The synaptic weight *W* (size of the PSC) can be modeled as a function of three different variables [2]:

(1) W=f(n,p,q).

Mechanisms acting on the number of release sites *n* seem to be targeted at long-term learning, while plasticity of the neurotransmitter release probability *p* and release quantity *q* both act on timescales of 0.1–1 seconds and are therefore well suited for extracting temporal fine structure of presynaptic pulse trains [3, 4]. Even for long-term learning, this short-term synaptic filtering may influence the type of learning [5]. Thus, dynamic synapses carry out various crucial signal transformations; for a review, see [3]. These transformations are used for processing sensory information, for example, in the auditory cortex [6].

Dynamic synapses also interact in a complex manner with another important component of neural information transmission, modulated pulse trains [7, 8], that is, spike trains characterized by regular shifts between high and low pulse rates [9]. In biology, these bursting spike trains have been implicated in the rapid transmission of information, encoding of stimuli, and population synchrony [7]. This interaction has been shown in simulations of models describing the plasticity of the synaptic release probability *p* [4] and also in models of the plasticity of the release quantity *q* [8, 10]. With regard to this interaction, it is often postulated as a general neural principle that a new stimulus is favorably transmitted over a steady-state one. This would mean that modulated spike trains, in which the stimulus continuously changes, would be favored over regular-rate stimuli. We will critically examine this assumption, extending the *q*-plasticity-based calculations of Natschlaeger and Maass [10].

The plasticity of *q* has been modeled in an influential manuscript by Markram et al. [11]. They introduced a formulation of quantal neurotransmitter release based on a descriptive model of biological mechanisms and measurements (in the following referred to as * quantal model *). Over the intervening years, the quantal model has been extensively studied with respect to its information transmission properties [3, 8, 10, 12]. It has also been combined with other synaptic plasticity mechanisms to investigate possible interrelations with long-term learning [3, 5] or probabilistic release models [8]. Various state-of-the-art neuroscience efforts still employ the original model, for example, in studies of pain reception [5], the differing modes of memory retrieval [13], or in the ongoing effort to fully characterize the model itself and its various processing characteristics [5, 13, 14]. Most of this work has been carried out via simulations, probably caused by the iterative, pulse-based nature of the model, making a closed solution, that is, some kind of transfer function, intractable. However, especially the causal dependency of the model's behavior on its parameters cannot be fully explained with simulations such as the ones in [10]. Rather, some kind of analytical expression is needed. This is especially interesting since biological synapses show very complex interdependences between their state variables and behavior [15, 16]; so an analytical expression of the biophysical model in [11] could be employed to identify the governing variables and mechanisms.

To derive this expression, we show that for regular pulse rates, the model by Markram et al. can be expressed explicitly as an exponential decay function. We use this function in Section 2 to deduce the response of a dynamic synapse to frequency modulated pulse trains. The veracity of the explicit expression is shown by comparison to simulations of the original quantal model in Section 3. Furthermore, we extend the optimality analysis of [10] to a wider parameter spectrum and give an explanation for the favored transmission of modulated spike trains in dynamic synapses.

## 2. Synaptic Transmission of Modulated Pulse Trains

### 2.1. Model of Activity-Dependent Synapses

The model developed by Markram et al. [11] is governed by two parameters, utilization of synaptic efficacy *u*_*n*_ and available synaptic efficacy *R*_*n*_. These are normalized as fractions of overall efficacy at pulse *n* of the pulse train. The model is based on a formulation of the refractoriness of neurotransmitter release, where available synaptic efficacy is dependent on the fraction used up in previous pulses. This increased usage is counteracted by a facilitation mechanism, which increases the utilization of synaptic efficacy (i.e., the available neurotransmitter amount) with rising pulse rate. Thus, utilization *u* is increased (facilitated) with each pulse and recovers with a time constant *τ*_facil_, while synaptic efficacy *R* recovers with *τ*_rec_, dependent on the current utilization. The iterative equations governing the evolution of *u*_*n*_ and *R*_*n*_ are as follows [11] (For *R*_*n*_, we use the index correction stated by Natschlaeger and Maass [10].):

(2) $$\begin{array}{cc} u_{n+1} & =u_{n}{\text{e}}^{-\Delta t_{n}/\tau_{\text{facil}}}+U\cdot \left(1-u_{n}{\text{e}}^{-\Delta t_{n}/\tau_{\text{facil}}}\right) \end{array}$$

(3) $$\begin{array}{cc} R_{n+1} & =R_{n}\left(1-u_{n}\right){\text{e}}^{-\Delta t_{n}/\tau_{\text{rec}}}+1-{\text{e}}^{-\Delta t_{n}/\tau_{\text{rec}}}, \end{array}$$

where Δ*t*_*n*_ denotes the time elapsed between pulses (*n* − 1) and *n* of the pulse train. The starting terms for (2) and (3) are computed from the utilization *U* of a relaxed synapse as *u*_1_ = *U* or *R*_1_ = 1 − *U*, respectively [11]. The PSC caused by a presynaptic pulse is defined as the product of *u*_*n*_ and *R*_*n*_, weighted with the absolute synaptic efficacy *A* (ratio between release quantity and resultant PSC):

(4) $$\text{PS}{\text{C}}_{n}=A\cdot R_{n}\cdot u_{n}.$$

The effect of this adaption can best be described as transmission of transients, that is, changes in the presynaptic pulse rate are transmitted with their full dynamic range to the postsynaptic neuron, but the response to steady-state input pulse rates diminishes. This seems to be a universal feature of biological neural nets, where novel stimuli receive increased responses compared to static ones [1, 3]. 

**Figure 1:**
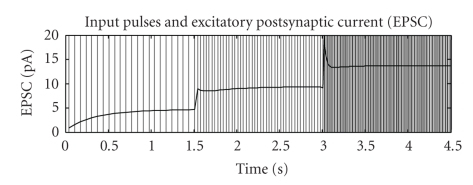
Behavior of the quantal synaptic short-term adaption, protocol similar to [11], Figure 4, but with regular pulse rates instead of Poisson, frequency step after 1.5 seconds and 3 seconds, pulse rates 15*s* ^−1^ → 30*s* ^−1^ → 80*s* ^−1^, the continuous curve denotes the resulting PSC. The parameters are identical to [11], Figure 4 , that is, *τ*_facil_ = 530 milliseconds, *τ*_rec_ = 130 milliseconds, A = 1540 pA, U = 0.03. To derive a continuous PSC from the pulse-PSC of (4), pulses with a duration of 1.4 milliseconds are weighted with the responses from (4), similar to the sum of PSCs as used in [10]. However, in contrast to [10], a moving average with a window of 100 milliseconds is computed to obtain a time curve rather than a scalar figure of merit. The pulse duration is not explicitly mentioned in [11], but biological evidence [1] and the similarity with [11] support this value.

For a steady-state signal, the above response can be thought of as a signal compression, so that the high dynamic range of, for example, sensory input is adapted to the limited range of the pulse response of a neuron [3]. The steady-state values that *u* and *R* settle to for a given pulse rate (Figure 1) can be computed by equating *u*_*n*_ and *u*_*n*+1_ in (2) for a fixed pulse rate *λ* [11]:

(5) $$u_{\text{c}}\left(\lambda \right)=\frac{U}{1-\left(1-U\right)\cdot {\text{e}}^{-1/(\lambda \cdot \tau_{\text{facil}})}}.$$

Using this *u*_c_ and a similar equalization approach, the convergent *R*_c_ is derived as

(6) $$R_{\text{c}}\left(\lambda \right)=\frac{1-{\text{e}}^{-1/(\lambda \cdot \tau_{\text{rec}})}}{1-\left(1-u_{\text{c}}\left(\lambda \right)\right)\cdot {\text{e}}^{-1/(\lambda \cdot \tau_{\text{rec}})}}.$$

As expected from the model, steady-state utilization *u* increases with higher pulse rate, whereas available synaptic efficacy *R* decreases. Due to the different time constants, these changes do not cancel out completely but lead to a maximum single PSC at around 20 Hz with slight decay for pulse rates below or above this value [11].

However, this steady-state analysis does not do justice to the complex transmission characteristics across a dynamic synapse. Consequently, in the following we analyze the response of a synapse to a single transient pulse rate transition. 

**Figure 2:**
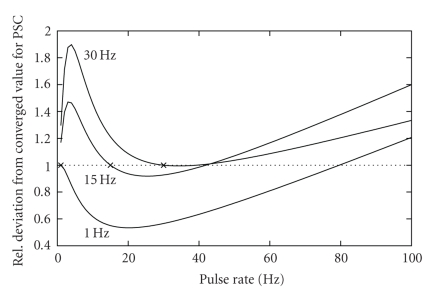
Relative PSC 5 Pulses after a step change in pulse rate. The synapse was in a converged state for a pulse rate of 1 Hz, 15 Hz, and 30 Hz, respectively. Then, the pulse rate was changed to the one denoted on the abscissa for 5 consecutive pulses. The mean PSC of the time window corresponding to the 5 pulses was calculated. This value was normalized to the mean PSC of a synapse being converged to the pulse rate after the step.

Figure 2  shows the response of the quantal synapse to a step change in pulse rate. The synapse starts out with one of three initial converged values for pulse rates 1 Hz, 15 Hz, and 30 Hz, respectively. We then transition to a new pulse rate as denoted on the abscissa for 5 consecutive pulses. The mean PSC of these 5 pulses after the step in pulse rate was normalised. The reference value for normalization is the converged PSC that would result from the steady-state PSC response for the pulse rate after the step, that is, the response if the new pulse train was not stopped after 5 pulses.

For decreasing pulse rate, the PSC response will continuously decrease, making the transient response bigger than the converged value. At first glance, one would expect the opposite for increasing pulse rate: if the PSC continuously increased, the transient PSC should be smaller than the converged value, and the quotient between both values should diminish for higher pulse rate differences because of *τ*_facil_ being bigger than *τ*_rec_ as well as the shorter time window. In contrast to that, Figure 2  shows transient PSCs higher than equilibrium for bigger step-ups in pulse rate; especially, for an initial 30 Hz rate, this is the case for all frequencies after the step change. This effect is caused by two processes: first, the time constant for utilization *u* considerably decreases with higher pulse rate, making it roughly equal to the time constant for efficacy *R* (see (A.7) in the appendix); second, the value for a single PSC decreases above a pulse rate of approximately 20 Hz [11], so that the resulting mean PSC sharply increases with the frequency step-up due to the higher number of releases per time but then is regulated down by the decreasing amplitude of a single PSC. This effect is also visible from the PSC time course in Figure 1.

As shown in this section, the response of dynamic synapses cannot be fully characterized by the transmission characteristics for regular pulse rates. The response for most cases of transients is amplified compared to the steady-state response. This is as expected from biology, where changes in pulse rate are a source of information, while static stimuli should be attenuated in favor of these transients [1, 3].

### 2.2. Analytical Approach to Synaptic Transmission

**Figure 3:**
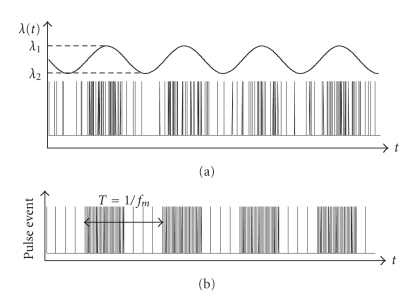
From top to bottom: bursty spike train, generated from a sine-modulated Poisson process and regularized approximation with rectangular modulation between high pulse rate *λ*_1_ and low pulse rate *λ*_2_ with a modulation frequency of *f*_*m*_.

In a generalization of the analysis carried out in Figure 2, in this section we derive the response of the quantal model to fully transient stimuli. Thus, we do not start from a converged value of *u* and *R* as in Figure 2, but we use repeating transient stimuli that result in regular variations in pulse rate (i.e., a modulated pulse signal; see Figure 3). 

A modulated pulse rate can be thought of as a sequence of bursts and as such represents a generic model for various types of neural pulse signaling, where the information is encoded in the temporal fine structure of the pulse signal [8, 9] or where bursts represent mechanisms in memory retrieval [13].

In the upper part of Figure 3, we generate a sine-modulated stochastic pulse train using a Poisson process [1] with time-variable pulse rate:

(7) $$f\left(\Delta t\right)=\lambda \left(t\right)\cdot {\text{e}}^{-\lambda (t)\cdot \Delta t},\;\Delta t\geq 0,$$

where *f*(Δ*t*) is the probability density function of the time between two successive spikes. In contrast to [1], we do not employ a fixed pulse rate *λ*, but one periodically sine-modulated between high pulse rate *λ*_1_ and low pulse rate *λ*_2_. We use this formulation for the simulations carried out in Section 2.3. However, for the mathematical analysis, we further simplify the stochastic bursting spike train in the upper part of Figure 3  to one that switches with a period of 1/*f*_*m*_ between two fixed pulse rates (see lower part of Figure 3). We additionally introduce a duty cycle *b* as the fraction of high rate stimulation per period. This enables a close approximation of different spiking modes (bursting, stuttering, etc.). For the approximation of the sine-wave, *b* = 0.5 is chosen.

**Figure 4:**
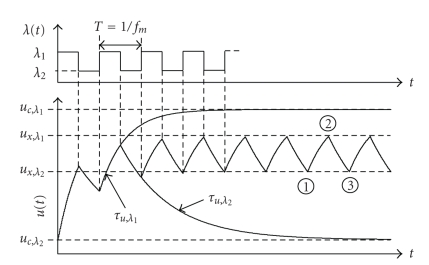
Time course of *u*(*t*) and its dependencies on modulation frequency *f*_*m*_ and convergence limits *u*_c_ for high and low pulse rates *λ*_1_ and *λ*_2_, respectively. *b* = 0.5 in this example.

Figure 4  qualitatively shows the time course of *u*(*t*) for this switched modulated stimulus. Its value oscillates inside a fixed amplitude interval [*u*_*x*,*λ*_2__, *u*_*x*,*λ*_1__] that depends on the modulation frequency *f*_*m*_, the duty cycle *b*, the convergence limits *u*_c_ for low and high pulse rate, as well as the time constants *τ*_*u*,*λ*_1__ and *τ*_*u*,*λ*_2__ defined by (A.7) in the appendix. 

For the derivation of the PSC's modulation dependency, we start with the explicit expression of (2) as derived in the appendix:

(8) $$u\left(t\right)=\left(u_{0}-u_{\text{c}}\right){\text{e}}^{-t/\tau_{u,\lambda }}+u_{\text{c}}.$$

Dependent on the sign of the term (*u*_0_ − *u*_c_), this equation describes one increasing or decreasing part of the time course, respectively. For a complete formulation, the initial values for each cycle must be calculated. These are generally not the limits of convergence, but intermediate values, as can be seen from Figure 4. Their calculation will be shown as an example for *u*_*x*,*λ*_2__ in the following. Our approach is based on the observation that the value of *u*(*t*) at points 1 and 3 in Figure 4  is the same in a steady-state. Following the time course of *u*(*t*) beginning at point 1 (assuming *t* = 0 there) gives

(9) $$\begin{array}{cc} u\left(t\right) & =\left(u_{x,\lambda_{2}}-u_{\text{c},\lambda_{1}}\right){\text{e}}^{-t/\tau_{u,\lambda_{1}}}+u_{\text{c},\lambda_{1}}\\ \to u\left(\frac{b}{f_{m}}\right) & =\left(u_{x,\lambda_{2}}-u_{\text{c},\lambda_{1}}\right){\text{e}}^{-b/(f_{m}\tau_{u,\lambda_{1}})}+u_{\text{c},\lambda_{1}}, \end{array}$$

with the second equation determining the value of *u*(*t*) at the end of the high rate interval. An analogous relation for the low-rate interval, that is, the time course from point 2 to 3, results in:

(10) $$u\left(\frac{1}{f_{m}}\right)=u_{x,\lambda_{2}}=\left[u\left(\frac{b}{f_{m}}\right)-u_{\text{c},\lambda_{2}}\right]{\text{e}}^{-(b-1)/(f_{m}\tau_{u,\lambda_{2}})}+u_{\text{c},\lambda_{2}}.$$

Evaluating (9) and (10) leads to the following expression for *u*_*x*,*λ*_2__

(11) $$u_{x,\lambda_{2}}=\frac{u_{\text{c},\lambda_{1}}{\text{e}}^{-(1-b)/(f_{m}\tau_{u,\lambda_{2}})}\left(1-{\text{e}}^{-b/(f_{m}\tau_{u,\lambda_{1}})}\right)+u_{\text{c},\lambda_{2}}\left(1-{\text{e}}^{-(1-b)/(f_{m}\tau_{u,\lambda_{2}})}\right)}{1-{\text{e}}^{-b/(f_{m}\cdot \tau_{u,\lambda_{1}})}\cdot {\text{e}}^{-(1-b)/(f_{m}\cdot \tau_{u,\lambda_{2}})}}.$$

Results for *u*_*x*,*λ*_1__, *R*_*x*,*λ*_1__ and *R*_*x*,*λ*_2__ can be derived with similar approaches.

Now, the mean synaptic release quantity $\bar{uR}=\bar{\text{PSC}}/A$ can be calculated. This is done by integrating the product *u*(*t*) · *R*(*t*), normalizing the result with the integration interval. For the high rate interval, that is, the time course between points 1 and 2, the following holds:

(12) $$\begin{array}{ll} {\bar{uR}}_{12} & =\frac{f_{m}}{b}\cdot \int_{0}^{b/f_{m}}\left[\left(u_{x,\lambda_{2}}-u_{\text{c},\lambda_{1}}\right){\text{e}}^{-t/\tau_{u,\lambda_{1}}}+u_{\text{c},\lambda_{1}}\right]\\ & \;\cdot \left[\left(R_{x,\lambda_{2}}-R_{\text{c},\lambda_{1}}\right){\text{e}}^{-t/\tau_{R,\lambda_{1}}}+R_{\text{c},\lambda_{1}}\right]\text{d}t. \end{array}$$

Evaluating this integral results in

(13) $$\begin{array}{ll} {\overset{¯}{uR}}_{12} & =\frac{f_{m}}{b}[\left(u_{x,\lambda_{2}}-u_{\text{c},\lambda_{1}}\right)\left(R_{x,\lambda_{2}}-R_{\text{c},\lambda_{1}}\right)\frac{\tau_{u,\lambda_{1}}\tau_{R,\lambda_{1}}}{\tau_{u,\lambda_{1}}+\tau_{R,\lambda_{1}}}\\ & \;\;\times \left(1-{\text{e}}^{-b(\tau_{u,\lambda_{1}}+\tau_{R,\lambda_{1}})/{(f}_{m}\cdot \tau_{u,\lambda_{1}}\cdot \tau_{R,\lambda_{1}})}\right)\\ & \;\;+\left(u_{x,\lambda_{2}}-u_{\text{c},\lambda_{1}}\right)\tau_{u,{\lambda \;}_{1}}R_{\text{c},\lambda_{1}}\left(1-{\text{e}}^{-b/\left(f_{m}\tau_{u,\lambda_{1}}\right)}\right)\\ & \;\;+\left(R_{x,\lambda_{2}}-R_{\text{c},\lambda_{1}}\right)\tau_{R,\lambda_{1}}u_{\text{c},\lambda_{1}}\left(1-{\text{e}}^{-b/{(f}_{m}\tau_{R,\lambda_{1}})}\right)\\ & \;\;+\frac{b\cdot u_{\text{c},\lambda_{1}}R_{\text{c},\lambda_{1}}}{f_{m}}]. \end{array}$$

Integrating over the low-rate interval, that is, the time course between points 2 and 3, in the same way yields the corresponding value ${\bar{uR}}_{23}$. 

As mentioned together with Figure 1, these mean values must be weighted by the number of pulses that occurred in the corresponding time interval. This can be done by using the ratio between the total time any pulse was active and the time interval:

(14) $$\begin{array}{ll} {\bar{\text{PSC}}}_{xy} & =A\cdot \frac{T_{\text{pulse}}N_{\text{pulse},x}}{T_{\text{norm}}}\cdot {\bar{uR}}_{xy}\\ & =A\cdot \frac{T_{\text{pulse}}\left(\lambda_{x}T_{\text{norm}}\right)}{T_{\text{norm}}}\cdot {\bar{uR}}_{xy}. \end{array}$$

For the high rate interval, *T*_norm_ = *b*/*f*_*m*_, whereas for the low-rate interval, *T*_norm_ = (1 − *b*)/*f*_*m*_. Using the corresponding constant pulse rate, *N*_pulse_ can be calculated for each interval as *N*_pulse,*x*_ = *λ*_*x*_ · *T*_norm_. For calculations, we will set *T*_pulse_ = 1.4 milliseconds, which is in agreement with the parameters used in [11].

When calculating an overall mean PSC, the duty cycle (i.e., the fraction each ${\bar{\text{PSC}}}_{xy}$ was active) has to be taken into account. This results in a weighted average formula:

(15) $$\overset{¯}{\text{PSC}}=b\cdot {\overset{¯}{\text{PSC}}}_{12}+\left(1-b\right)\cdot {\overset{¯}{\text{PSC}}}_{23}=AT_{\text{pulse}}\cdot \left(b\lambda_{1}{\overset{¯}{uR}}_{12}+\left(1-b\right)\lambda_{2}{\overset{¯}{uR}}_{23}\right).$$

### 2.3. Results

**Figure 5:**
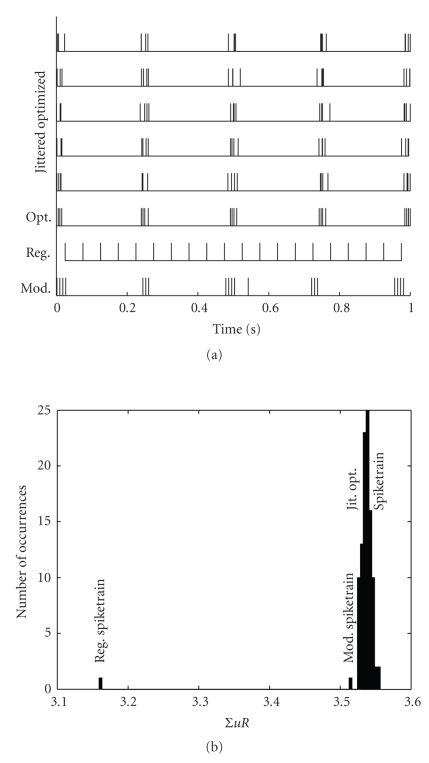
Left: spike trains as applied to the original quantal model, from bottom to top: high (100 Hz) and low (2 Hz) frequency modulated spike train (*f*_*m*_ = 4.2 Hz, *b* = 0.12), regular spike rate (20 Hz), spike train as extracted from Figure 5(a)  in [10] (*U* = 0.15, *τ*_rec_ = 144 milliseconds, *τ*_facil_ = 62 milliseconds), and five jittered versions of this spike train (*σ* = 5 milliseconds). Right: histogram of the sum over the product *uR*, with the data points for modulated and regular spike train indicated.

The explicit expressions derived in Section 2.2 describe the behavior of PSC transmission dependent on the modulation frequency. To evaluate these equations, we compare our model to numerical simulations of the original iterative equations (2) and (3). In particular, Natschlaeger et al. [10] treat the quantal model to a rigorous numerical analysis; so we apply our model to their framework. Since the optimal spike trains of [10] differ from our modulated pulse rate assumption, we have to validate that the sum over the product *uR*, that is, the PSC efficacy criterion, has the same quantitative and qualitative behavior for the modulated rate as for the optimized spike train. An initial validation can be done by extracting a sample spike train for a single parameter set from [10], applying a jitter to account for extraction errors, and comparing it to a modulated spike train which is parameterized to exhibit a similar burstiness. This is shown in Figure 5. 

The parameters were chosen to resemble the experiment of Figure 5  in [10], with 20 pulses distributed in a one-second interval. All spike trains were processed with the original quantal model. As can be seen, the original optimized spike train shows a strong burstiness, so as expected, the regular spike train has a much lower synaptic efficacy. Also, the modulated spike rate is well within bandwidth of the statistical variations of the optimized spike train and also shows significantly larger synaptic efficacy than the regular rate. From this limited example (and others below), the initial assumption for our derivation seems valid, that is, a modulated pulse rate exhibits the same behavior with respect to the Markram model as a more precisely optimized one.

In the following, we will thus apply the derivation of Section 2.2, in particular the new non-iterative time constants, to extend the analysis of [10] and especially test the predictive and explanatory power of our analytical expressions. Two major activity regimes can be discerned from Figure 5  of [10]: one where the grouping of pulses into short activity bursts results in a large synaptic efficacy, and one where in contrast a regular distribution of all 20 pulses across the time interval is advantageous. If we relate this back to our model, the pulse regime is determined by the modulation frequency. Thus, with the explicit expression for the mean PSC (15), we can state an alternative optimality approach to [10]. Maximizing the mean PSC over the modulation frequency corresponds to finding the optimum pulse regime for a synapse. The optimum modulation frequency *f*_*m*,opt_ in that sense can be derived using the necessary condition:

(16) $$0=\frac{\partial \overset{¯}{\text{PSC}}}{\partial f_{m}}=AT_{\text{pulse}}\cdot \left(b\lambda_{1}\frac{\partial {\overset{¯}{uR}}_{12}}{\partial f_{m}}+\left(1-b\right)\lambda_{2}\frac{\partial {\overset{¯}{uR}}_{23}}{\partial f_{m}}\right).$$

In general, this equation cannot be brought in an explicit form. Approximate explicit expressions could be derived, for example, assuming *f*_*m*_ *τ*_*u*,*λ*_ ≫ 1, but the respective approximations are not valid over the entire synapse parameter space and optimization range. Thus, we will solve the optimization equation numerically. Modulated (i.e., bursty) spike trains are only generated in the *f*_*m*_-range (0, 1/2*λ*_1_); other values result in a regular spike train. Thus, if the partial derivative $\partial \bar{\text{PSC}}/\partial f_{m}$ does not change sign inside this interval, that is, no local maximum exists therein, a regular spike train will result in a maximum mean PSC. 

**Figure 6:**
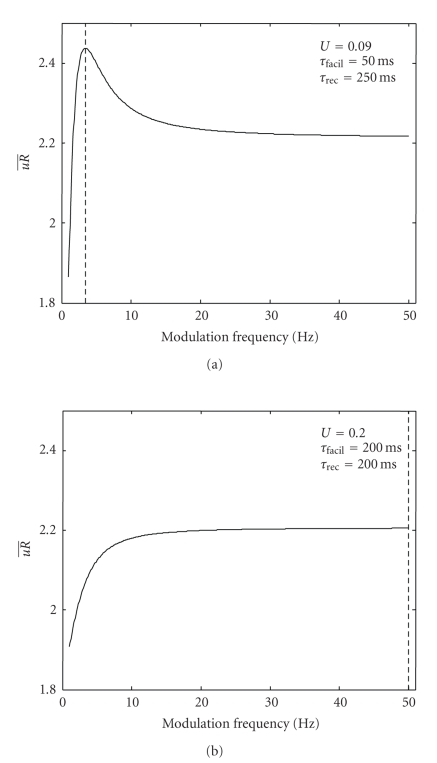
Mean synaptic efficacy per spike $\bar{uR}$ with respect to modulation frequency *f*_*m*_ for two parameter sets. Dashed line: optimum modulation frequency for the plotted range. *λ*_1_ = 100 Hz, *λ*_2_ = 5 Hz in both cases.

To resemble the optimization regime of [10], we adjust the duty cycle *b*(*λ*_1_, *λ*_2_) such that the mean frequency $\bar{f}=20$ Hz. The results of [10] show that a modulated regime is optimal for low values of *U* and *τ*_facil_, whereas for higher values, a regular spike train is favorable. Figure 6  confirms this result with our analysis for an illustrative example: for the low-value case (left), a maximum at approximately 4 Hz is present, whereas for the high-value case, the synaptic efficacy monotonically increases with modulation frequency, which ultimately leads to a regular spike train as an optimum. 

Of course, as we have shown in the previous section and the appendix, the preference of the quantal model depends not only on *U*, *τ*_facil_, and *τ*_rec_ but also on the spike train characteristics, that is, duty cycle *b*, high rate *λ*_1_, and low-rate *λ*_2_. To show these dependencies, we extend the analysis of Figure 6  to a full sweep across *b*, *λ*_1_ and *λ*_2_, employing the synapse parameter set of Figure 6, left. 

**Figure 7:**
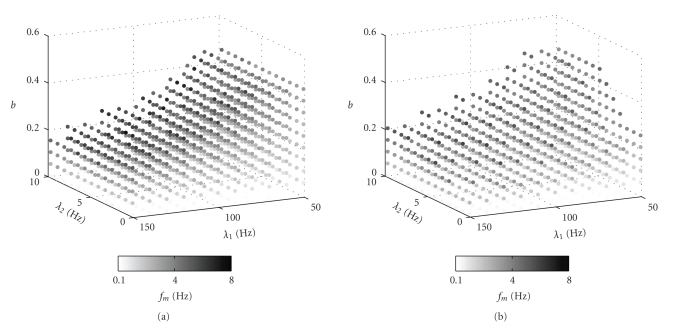
Optimal modulation frequency *f*_*m*,opt_ (grey scale coded) with respect to the parameters of the modulated spike train. (a) simulation, and (b) our analytical calculation. Only those cases are shown where a modulated spike train is favored. Parameters as in Figure 6, left.

Figure 7  shows the optimal modulation frequency *f*_*m*,opt_, derived similar to Figure 6, in grey-scale. Data points are only depicted if a distinct optimal *f*_*m*_ is found, that is, if the maximum as shown in Figure 6, left, is at least 1% above the value of $\bar{uR}$ for the high modulation frequency (right side of both graphs in Figure 6). Thus, nonsignificant maxima and cases where a regular spike train is preferred (Figure 6, right) are omitted. A good correspondence between the simulation of the original quantal model and the mean $\bar{uR}$ as derived from the analysis in the previous section can be observed, showing the validity of our derivations. 

There is almost no dependence of the optimal modulation frequency and the burst preference on the low spike rate *λ*_2_. This may be due to the fact that there is only a certain level of relaxation that can be obtained by the synapse during the low-rate intervals. This means that, while the relaxation is important to obtain a high $\bar{uR}$, as will be explained together with Figure 9, the exact low rate during this relaxation is not important, only the fact that there is such a relaxation phase. However, there is a clear dependence between the duty cycle *b* and *f*_*m*_, where *f*_*m*_ rises linearly with *b* at a certain *λ*_1_, *λ*_2_ (see columns in the plots of Figure 7). In other words, this can be thought of as

(17) $$\frac{b}{f_{m}}=\text{const}.=b\cdot T=T_{\text{high}}\;,$$

with *T* being the duration of a period and *T*_high_ being the duration of the high rate interval therein, that is, the length of a burst. Thus, if *T*_high_ is constant, the number of pulses during a burst for a given high rate *λ*_1_ is also constant. An explanation for this could be that there exists an optimal burst profile which maximizes $\bar{uR}$ for a given parameter set *U*, *τ*_facil_, *τ*_rec_, and a given *λ*_1_. Accordingly, if *b* is subjected to a sweep, *f*_*m*_ must rise with it to keep this optimal profile. At the same time, bursts are shifted closer, so that the mean number of pulses in a fixed time interval rises linearly with *b*. Equation (16) thus searches not so much for an optimal *f*_*m*_ but rather for an optimal burst profile.

Another interesting characteristic of the above plot is the decrease of the maximum *b* at which a significant *f*_*m*,opt_ can be found with increase in *λ*_1_. This inverse relationship between maximum *b* for a bursty spike train and *λ*_1_ may hint at an optimal profile or number of pulses in a burst almost independent of *λ*_1_. According to (17), the number of spikes in a burst can be computed as *b*/*f*_*m*_ · *λ*_1_, resulting in a (mean) number of pulses per burst of 4.3 for *λ*_1_ = 150 Hz and 3.6 for *λ*_1_ = 50 Hz. These similar values could be explained by the fact that the optimum $\bar{uR}$ is governed by the evolution of *u* and *R* during the burst. These in turn depend on the absolute time constants derived in the appendix, which scale with *λ*; thus, the scaling of the time constants and *λ* cancel each other at least partially, resulting in very similar optimal burst profiles despite the change in *λ*_1_. So the absolute value of $\bar{uR}$ may vary with *λ*_1_, *b* and *f*_*m*_, but the qualitative behavior, that is, the burst profile for which $\bar{uR}$ is maximum, seems to be constant for a given synapse type. Interestingly, there exists no optimal modulation frequency above 8Hz, that is, in this range a regular spike train is always better than a modulated one. This is probably due to the fact that at this *f*_*m*_, there is a natural transition between bursty and regular spike train in any case. That means, the burst phases are too short to allow a real grouping of spikes, while the low-rate phases are too short to obtain a significant recovery of *u* and *R*, so that the same number of pulses achieves a higher $\bar{uR}$ if it is spaced regularly across the given time span. 

**Figure 8:**
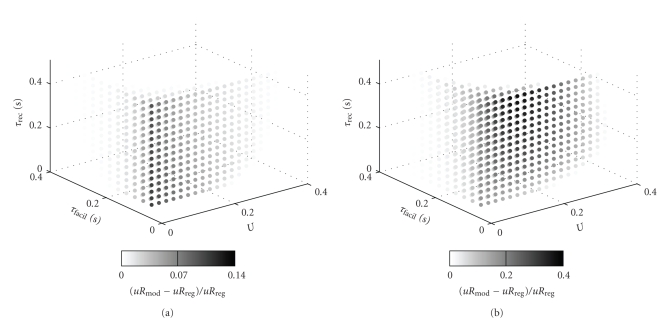
Relative difference of a modulated spike train from a regular spike train, grey scale coded, with respect to the parameters of the quantal model. (a) simulation, and (b) our analytical calculation. Only positive (i.e., modulation-favored) part is shown.

As already stated, one of the main questions behind such analyses is, for which synapse types (i.e., parameters *U*, *τ*_facil_ and *τ*_rec_) a modulated spike train is favored over a regular spike train in terms of transmission. This question was tackled in [10] only exemplarily for single-value sweeps. Here, we perform a sweep over the full three-dimensional parameter space of the quantal model, as shown in Figure 8. Thereby we use the relative difference of a modulated spike train and a regular spike train as a measure for the favored spike mode. Parameters were again *λ*_1_ = 100 Hz, *λ*_2_ = 5 Hz, and $\bar{f}=20$ Hz, together with a modulation frequency *f*_*m*_ = 8 Hz, comparing to results of Figure 5  in [10]. 

We also used this parameter sweep to compare our analytical calculation with the original iterative formula. This is a hard test case, because already small deviations in the calculations, for example, caused by the continuous-time idealization and the approximations made with the derivation of *τ*_*R*,*λ*_, can lead to marked changes in the relative difference value calculated for comparison. Taking this sensitivity into account, our derivation is in good agreement with the simulation. Especially the discrimination between favored modulated or regular spike train is well replicated.

The principal dependencies of the favored spike mode on the synapse parameters as suggested by Figure 5  of [10] are also present in the whole parameter space exploration: A modulated spike train is only favored if *U* or *τ*_facil_ are low. Also, for *U* = 0.32 and *τ*_facil_ = 62 milliseconds, a transition from regular-favored to modulation-favored transmission with increasing *τ*_rec_ is present in the plot, which is in agreement with [10]. This again shows that even with the assumption of a fixed modulated, and potentially nonoptimal, spike train, essentially the same predictions can be derived as with a single-spike optimization, but with much less computational complexity. Also, further dependencies can be extracted from the parameter sweep: if both *U* and *τ*_facil_ decrease to low values, the relative difference of the response to modulated and regular spike trains gets more and more independent of *τ*_rec_. Additionally, to a certain extent, higher values for *U* can be compensated with lower values for *τ*_facil_, and vice versa.

In the following, we try to analyze the above parameter dependencies, based on our modeling of *u* and *R* as exponential decays. In particular, based on our noniterative time constants for *u* and *R* and on the converged values *u*_*c*_ and *R*_*c*_ that scale the exponential decay functions, we postulate the following mechanisms for a preference of either regular or grouped (bursty) spike trains by the synapse.

**Figure 9:**
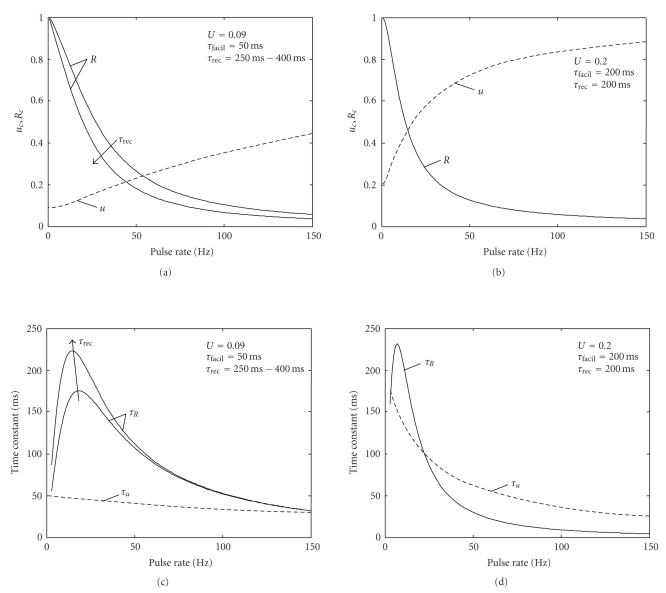
(a) Steady-state values of *u* and *R* over spike rate for a bursty parameter set, with additional indication of R dependency on *τ*_rec_ (b) same as (a) for a regular parameter set (c) convergence time constants for the parameter set(s) of (a) (d) convergence time constants for the parameter set of (b).

There is a dependency of this preference on the relation between the time constants *τ*_*R*,*λ*_1__ and *τ*_*u*,*λ*_1__, that is, for the convergence during the high rates/bursts (Figure 9(c)). A bursty spike train benefits if the time constant *τ*_*u*,*λ*_1__ is relatively low, so that *u* rises fast to its converged value, which is a factor of five above its relaxed value (i.e., at the end of the low-rate interval, Figure 9(a)), as this increases markedly the total value of *uR*. On the other hand, R diminishes to a small value for the high rate, so its convergence time constant *τ*_*R*,*λ*_1__ should be large relative to *τ*_*u*,*λ*_1__, so that most of the spikes during the burst still “see” the high relaxed value (Figure 9(a)). Compared to a parameter regime which preferentially transmits a regular rate (Figure Figure 9(b)  and 9(d)), a low time constant *τ*_*R*,*λ*_1__ diminishes the value of *R* during a burst, and a corresponding high time constant *τ*_*u*,*λ*_1__ would prevent *u* from rising to compensate this decrease in *R*, especially if at the same time *u* has a smaller dynamic range (Figure  9(b)). Thus, for this parameter regime and its resulting time constants, a bursty regime would result in a lower synaptic efficacy compared with a regular rate. 

A second criterion based on which it can be predicted if a bursty or regular regime is preferred by the synapse, would be the relation between the convergence time constant for *R* for low and high rate *τ*_*R*,*λ*_2__ and *τ*_*R*,*λ*_1__. This relation expresses the basic intuition that *τ*_*R*,*λ*_2__ should be relatively low compared to *τ*_*R*,*λ*_1__, so that *R* can relax very fast to a high value during the low-rate intervals. In contrast, *τ*_*R*,*λ*_1__ should be high compared to the burst duration so that R does not decrease too much during the high rate intervals. So a parameter regime that results in a low *τ*_*R*,*λ*_2__ relative to *τ*_*R*,*λ*_1__ should preferentially transmit bursts.

**Figure 10:**
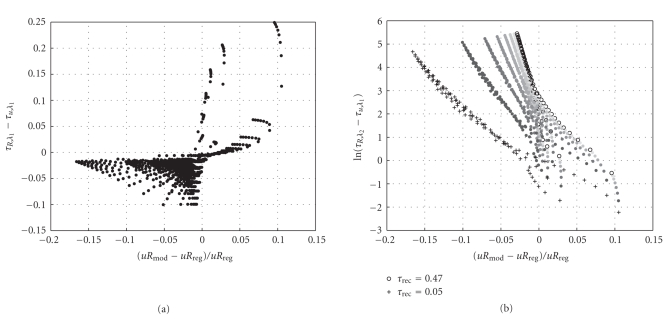
Correlation between criteria based on the convergence time constants of (A.12) and (A.7) (y-axes) and the relative difference between the synaptic efficacy *uR* for a regular and a modulated/grouped spike train (x-axes). Each of the dots corresponds to one data point from Figure 8, that is, one parameter set (*U*, *τ*_facil_, *τ*_rec_). In addition, the right figure has a decrease in *τ*_rec_ denoted by increasing gray levels. Also, the smallest (resp. largest) value for *τ*_rec_ is denoted in crosses resp. circles.

From the above postulates, two criteria can be derived where the time constants derived in this paper allow to predict if a grouped/bursty or a regular regime is preferred by the synapse. The first one would be the difference between the convergence time constants for the high rates, that is, *τ*_*R*,*λ*_1__ − *τ*_*u*,*λ*_1__, see Figure 10(a). 

As can be seen, there is a definite correlation in the way suggested above, that *τ*_*R*,*λ*_1__ should be larger than *τ*_*u*,*λ*_1__ in order for the synapse to transmit a bursty spike train better than a regular one. In the figure, this is expressed on the x-axis by the normalized difference between Σ*uR* for a regular, respectively, a bursty spike train (for the same quantal parameter set). The second criterion would be the quotient between the convergence time constant for *R* for low and high rate, as expressed by *τ*_*R*,*λ*_2__/*τ*_*R*,*λ*_1__. Figure 10(b) shows a plot of this criterion, against the same synaptic efficacy criterion as in Figure 10(a). For clarity reasons, the natural logarithm of the above quotient is plotted rather than the quotient itself. Again, as postulated above, there is a clear correlation between a measure based on the convergence time constants and the amount a bursty spike train evokes more or less synaptic efficacy compared to a regular spike train. Interestingly, there also seems to be some parameter which causes a change in slope as well as a shift of the correlation curve. When plotting the data points based on their parameter values, it becomes evident that this parameter is *τ*_rec_, that is, for larger *τ*_rec_, the spike trains enter the bursty regime earlier. This trend towards burstiness with increasing *τ*_rec_ can be explained based on Figures 9(a)  and 9(c). As can be seen, for larger *τ*_rec_ the slope of the *R* curve increases, so that the (converged) value of *uR* for a regular rate diminishes, while for the short high rate episodes characteristic of a burst, the relaxed *R* is still close to one. Due to the fact that *τ*_*u*,*λ*_ for the high rate does not decrease, the burst benefits from this high relaxed value in the same way as it did for lower *τ*_rec_. At the same time, *τ*_*R*,*λ*_ increases with increasing *τ*_rec_, so that there is a more pronounced trend towards long periods of little activity in the spike train, so that *R* can reach its relaxed value even if its convergence time constant becomes larger. That the synapse exhibits a mechanism which prefers modulated spike trains for certain parameter sets (as shown above) might also provide an alternative, synapse-based way for bursting behavior to emerge. This could complement the conductance-based bursting behavior shown in [7].

How could these results be applied in the wider neuroscience context? One important topic of current interest is the interaction of the different forms of plasticity on the same synapse, especially with regard to the different temporal timescales of expression [5, 15–17]. Some studies which employ both kinds of plasticity act on an abstract idea of weight, but with basically unchanging parameters of the dynamic synapse [5]. On the other hand, Spike-Timing-Dependent Plasticity (STDP) is postulated to depend on the modulation of neurotransmitter release probability ([16, 17]), which in the model discussed herein is expressed as the initial release quantity *U* [11]. As evidenced by our analysis, this influences directly the spike pattern preferences through the mechanisms postulated in Figure 9. So these are not just plasticity mechanisms overlaid on the same synaptic weight [17], instead STDP might govern the operating regime of the short term dynamics. Thus, STDP might not only provide a basis for static, weight-based memory formation [18], but also serve as a substrate for memory and computation in dynamical models. Examples for this could be attractor neural networks [13], or liquid computing [19], which rely heavily on the short term dynamics of synapses. In this respect, our analysis indicates several ways in which a *U* modulated by some other plasticity mechanisms might in turn govern the absolute temporal dynamics of a synapse, namely through *u*_*c*_, *τ*_*u*,*λ*_, and *τ*_*R*,*λ*_. Pushing this speculation further, there might also be a feedback path back towards STDP, in which the absolute synaptic time constants *τ*_*u*,*λ*_ and *τ*_*R*,*λ*_ of our derivation influence the time course of the STDP learning window. Of course, classical STDP relies on coincidence between pre- and postsynaptic spikes, so the quantal release mechanism which only acts on presynaptic spikes would not work in this context. However, several newer forms of STDP rely on dendritic spikes [20], which depend on coincident heterosynaptically expressed presynaptic spike transmission rather than on postsynaptic spikes. Thus, this form of STDP could, through its influence on *U*, change *τ*_*u*,*λ*_ and *τ*_*R*,*λ*_ and these in turn would impact on the temporal learning window. This could form some kind of metaplasticity or homeostasis [21], in which STDP influences its own expression at the synapse.

## 3. Conclusion

We have derived an explicit expression for the iterative quantal model [11] describing short term plasticity of dynamic synapses. A wide range of naturally occurring pulse trains could be subjected to detailed mathematical analysis using this model. For example, our analysis is also valid if the pulse rate during a burst is not constant (see Figure 5). Thus, the selective treatment of bursts by dynamic synapses as derived in Section 2 could also be extended to cases were the information is contained in the fine structure of the bursts [4, 8, 12]. Also, the modulation does not have to be constant, that is, pauses between bursts could vary, so that pulse trains derived in [8, 14] could also be treated with a more rigorous, global approach, rather than an analysis via simulations.

We have shown how the filtering characteristics might be determined from the synaptic parameters. Specifically, we have provided an explanation how the filtering characteristic of a dynamic synapse depends on the effective time constants *τ*_*u*,*λ*_ and *τ*_*R*,*λ*_ and their interaction with the converged values for *u* and *R* (see Figures 5  and 10). Also, in extension of [10], we have provided a more complete picture how the filtering characteristic relates back to the original parameters (see Figure 8). The mechanisms/correlations shown in Figure 10  could be applied to characterize the transfer/decoding function of synaptic networks, such as the ones used in [4]. Also, the closed expression for the transfer function developed in this manuscript could be employed to deriving the synaptical parameter set for the optimal coding of stimuli in, for example, the auditory cortex [6]. We have shown a limited example of this in Figure 6, where we use our transfer expression to derive the optimal modulation frequency for a parameter set, which is in good agreement with the numerical simulations of [10]. 

Our noniterative expression for the behavior of the dynamic synapses of [11] could also have consequences on plasticity mechanisms. Synapses exhibit very diverse modulatory and plastic behaviors, where the interdependences and governing variables often cannot be clearly determined [15, 16]. Since the time constants of neural actions are not very amenable to change [1], it might be assumed that the temporal dynamics and preferences of a synapse are relatively fixed. In contrast, our derivations in this paper predict mechanisms by which a synapse could change its effective time constants and spike pattern preference based on *U* even though the basic temporal parameters *τ*_rec _ and *τ*_facil_ of the dynamic synapse might be constant. In this context, we have speculated on possible repercussions of this modulation of *τ*_*u*,*λ*_ and *τ*_*R*,*λ*_ on models of long-term plasticity (STDP), especially with regard to extending STDP to dynamical models of computation and learning/memory.

## Figures and Tables

**Figure 11:**
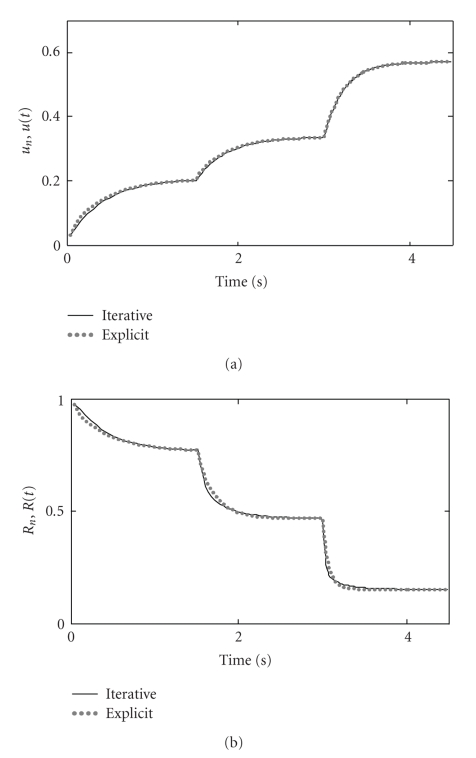
Comparison of simulated and analytically derived time course of *u* (A) and *R* (B). Parameters are the same as used in Figure 1.

## Appendix

### A. Transient Analytical Description of Quantal Plasticity

The convergence of iterative equations like those of the quantal model [11] can only be expressed explicitly for some special cases [22]. Whereas the convergence limits for a constant presynaptic pulse rate *λ* can be derived with relatively little effort [11], the time course and speed of convergence is difficult to define, especially due to the constant parts of the iterative equations for *u*_*n*_, (2), and *R*_*n*_, (3).

However, as Figure 11  shows, in case of regular spike trains with a defined pulse rate, the iterative descriptions of *u* and *R* in (2) and (3) can be interpreted as settling of transient responses to a steady-state value, comparable to the exponential convergence of, for example, an RC voltage settling curve.

In this case, an absolute time constant for this settling may be derived, which is likely to depend on the fundamental time constants of the quantal model.

In the following, an explicit expression for the convergence of *u* toward a steady-state value will be derived. Equation (2), recursively describing the value of *u* after inter-spike interval (ISI) Δ*t*_*n*_, can be rewritten as

(A.1) $$u_{n}=u_{n-1}\cdot {\text{e}}^{-\Delta t_{n-1}/\tau_{\text{facil}}}\cdot \left(1-U\right)+U.$$

Note that all variables are shifted by one ISI compared to the original formulation. For deriving an explicit expression, we restrict ourselves to pulse trains having a constant rate *λ*, so that Δ*t*_*n*_ = 1/*λ* for all *n*. Recursively extending (A.1) by one ISI yields

(A.2) $$u_{n}=u_{n-2}\cdot {\text{e}}^{-2/\left(\lambda \cdot \tau_{\text{facil}}\right)}\cdot {\left(1-U\right)}^{2}+U\cdot {\text{e}}^{-1/(\lambda \cdot \tau_{\text{facil}})}\cdot \left(1-U\right)+U.$$

The further recursion back to *u*_0_ is obvious from (A.2), resulting in

(A.3) $$u_{n}=u_{0}\cdot {\left[(1-U){\text{e}}^{-1/\left(\lambda \cdot \tau_{\text{facil}}\right)}\right]}^{n}+U\cdot \overset{n-1}{\underset{i=0}{\sum }}{\left[(1-U){\text{e}}^{-1/\left(\lambda \cdot \tau_{\text{facil}}\right)}\right]}^{i}.$$

Because the term (1 − *U*)e^−1/(*λ*·*τ*_facil_)^ never exceeds the interval [0, 1), the geometric series of the second term converges, and its sum can be calculated to yield [22]:

(A.4) $$u_{n}=u_{0}\cdot {\left[(1-U){\text{e}}^{-1/(\lambda \cdot \tau_{\text{facil}})}\right]}^{n}+U\frac{{\left[(1-U){\text{e}}^{-1/(\lambda \cdot \tau_{\text{facil}})}\right]}^{n}-1}{\left(1-U\right){\text{e}}^{-1/\left(\lambda \cdot \tau_{\text{facil}}\right)}-1}.$$

The limit for *n* → ∞ is the same as the value for *u*_c_(*λ*) calculated in (5). In fact, equation (A.4) can be rewritten in the following form to make this limit obvious:

(A.5) $$u_{n}=\left(u_{0}-\frac{U}{1-\left(1-U\right){\text{e}}^{-1/\left(\lambda \cdot \tau_{\text{facil}}\right)}}\right)\cdot {\left((1-U){\text{e}}^{-1/\left(\lambda \cdot \tau_{\text{facil}}\right)}\right)}^{n}+\frac{U}{1-\left(1-U\right){\text{e}}^{-1/\left(\lambda \cdot \tau_{\text{facil}}\right)}}.$$

The speed of convergence is determined by the term dependent on *n*. For introducing the notion of a time constant, we extend *u*_*n*_ in (A.5) to a continuous-time variable *u*(*t*) that is equal to *u*_*n*_ at the time of pulse *n*, which means *u*(*n* · Δ*t*) = *u*_*n*_ for a constant pulse rate. At this point in time, equality *n* = *λ* · *t* holds, which we use to reformulate the term dependent on *n*:

(A.6) $${\left((1-U){\text{e}}^{-1/\left(\lambda \cdot \tau_{\text{facil}}\right)}\right)}^{\lambda \cdot t}={\text{e}}^{t\cdot \left[\lambda \cdot \ln\;(1-U)-1/\tau_{\text{facil}}\right]}={\text{e}}^{-t/\tau_{u,\lambda }},$$

with the time constant describing the speed of convergence following:

(A.7) $$\tau_{u,\lambda }=\frac{1}{\lambda \cdot \ln\;\left(1/\left(1-U\right)\right)+1/\tau_{\text{facil}}}.$$

The time constant thus is dependent on both the time constant of the iteration, *τ*_facil_, and the pulse rate, *λ*. Therefore, (A.5) can be modeled as follows:

(A.8) $$u\left(t\right)=\left(u_{0}-u_{\text{c}}\right){\text{e}}^{-t/\tau_{u,\lambda }}+u_{\text{c}}\;.$$

An explicit expression for *R*_*n*_ can be derived in a similar way, starting with an equation analogous to (A.2):

(A.9) $$R_{n}=R_{n-2}\cdot \left(1-u_{n-2}\right)\left(1-u_{n-1}\right){\text{e}}^{-2/\left(\lambda \cdot \tau_{\text{rec}}\right)}+\left(1-{\text{e}}^{-1/\left(\lambda \cdot \tau_{\text{rec}}\right)}\right)\cdot \left[\left(1-u_{n-1}\right){\text{e}}^{-1/\left(\lambda \cdot \tau_{\text{rec}}\right)}+1\right].$$

This again makes the further recursion back to *R*_0_ clear:

(A.10) $$R_{n}=R_{0}\cdot {\text{e}}^{-n/\left(\lambda \cdot \tau_{\text{rec}}\right)}\overset{n-1}{\underset{i=0}{\prod }}\left(1-u_{i}\right)+\left(1-{\text{e}}^{-1/\left(\lambda \cdot \tau_{\text{rec}}\right)}\right)\times \{\overset{n-1}{\underset{j=0}{\sum }}\left[{\text{e}}^{-(n-j)/\left(\lambda \cdot \tau_{\text{rec}}\right)}\cdot \overset{n-1}{\underset{i=j}{\prod }}\left(1-u_{i}\right)\right]+1\}.$$

Because of the varying *u*_*i*_, the terms in the product are not constant like in the derivation for *u*, so that the resulting series is not geometric, making a straightforward derivation impossible. Nevertheless, an exponential decay with a fixed time constant like for *u* is the dominant behavior also for *R*, as can be seen from Figure 11. We will therefore use a heuristic approximation for deriving an explicit expression for the time constant of *R*. We first insert the explicit expression of *u*_*n*_ (A.8) and the starting value for *R*_0_ to derive the following expression:

(A.11) $$\begin{array}{ll} R_{n} & =\left(1-{\text{e}}^{-1/\left(\lambda \tau_{\text{rec}}\right)}\right)\\ & \;\cdot \{\overset{n-1}{\underset{j=0}{\sum }}[{\left(-\frac{M}{1-M}\cdot {\text{e}}^{-1/(\lambda \tau_{\text{rec}})}\right)}^{j}\\ & \;\;\;\;\;\times \overset{n-1}{\underset{i=n-j}{\prod }}\left(1-{\text{e}}^{-1/(\lambda \tau_{\text{facil}})}-U\cdot M^{i}\right)]+1\},\\ & \;\text{with}\;\;\;\;\;M=\left(1-U\right)\cdot {\text{e}}^{-1/\left(\lambda \tau_{\text{facil}}\right)}. \end{array}\;\;$$

The constant factor outside the sum does not have an influence on the convergence speed of *R*. When neglecting the product inside the sum, the expression reduces to the sum of a geometric series, whose time constant may be determined analogously to (A.4) and (A.5). The deviations from the original formulation then have to be corrected by additional or modified terms in the time constant. The exponential function and the term for *M* in the numerator of the simplified expression result in terms 1/*τ*_rec_ and 1/*τ*_facil_ in the time constant, respectively. By using the approximation (1 − *ε*) = e^*ε*^, the term (1 − *M*) in the denominator can be transformed to yield an inverse proportional relation between *U* and the time constant for *R*, *τ*_*R*,*λ*_.

Now, the product term in (A.11) has to be accounted for. The term *M* ^*i*^ results in a quadratic dependency between the time constant for *R* and the pulse rate *λ* due to the additional potentiation of *M*. At the same time, the term (1 − *U*) of *M* ^*i*^ is again transformed into ln 1/(1 − *U*), resulting in *λ* ^2^ *τ*_facil _ ln 1/(1 − *U*). The constant factor *U* results in an inverse linear dependency of *τ*_*R*,*λ*_ on *λ*, while *U* is again transformed into ln 1/*U*. The term (1 − e^−1/(*λτ*_facil_)^) can be neglected for high frequencies *λ*, but has to be taken into account for low frequencies. This can be done by combining the constant terms 1/*τ*_rec_ and 1/*τ*_facil_ while adding a frequency-dependent factor *U* · *λ*.

With all principal dependencies being identified, the resulting time constant of *R* reads:

(A.12) $$\begin{array}{cc} \tau_{R,\lambda } & =\frac{1}{U\cdot \left[\lambda^{2}\cdot \tau_{\text{facil}}\cdot \ln\;(1/\left(1-U\right))+2/3\lambda \cdot \ln\;(1/U)+2/(3\tau_{\text{rec}}\cdot U\tau_{\text{facil}}\cdot \lambda )\right]}, \end{array}$$

Analogously to the derivation for *u*, an explicit formulation for *R* can be stated using the time constant defined by equation (A.12):

(A.13) $$R\left(t\right)=\left(R_{0}-R_{\text{c}}\right){\text{e}}^{-t/\tau_{R,\lambda }}+R_{\text{c}}.$$

Equations (A.8) for *u* and (A.13) for *R* were verified against simulations of the iteration formulae (2), (3) over a wide range of parameters for *τ*_facil_, *τ*_rec_ and *U*, see results. Despite the continuous-time generalization and the approximations made in the derivation of *τ*_*R*,*λ*_, analytical and simulation results are in good agreement. Figure 11  shows an example of the resulting time courses. The differences between simulated and analytically derived *u*(*t*) are due to the discrete nature of the original iterative equations that were generalized to continuous time for the analytical formulation. Slightly bigger, but still negligible deviations are visible for *R*(*t*), which are due to the approximations made.
